# Implantable Cardiac Defibrillator-Related Culture-Negative Infection: A Case of *Coxiella burnetii* Infection

**DOI:** 10.3390/jcm12082817

**Published:** 2023-04-11

**Authors:** Panteleimon E. Papakonstantinou, Victoria Georgiadou, Grigorios Doumanis, Vasiliki Bistola, Joseph Papaparaskevas, Gerasimos Filippatos, Sotirios Xydonas

**Affiliations:** 1 2nd Cardiology Department, Evangelismos Hospital, 106 76 Athens, Greece; 2 2nd Cardiology Department, “Attikon” University Hospital of Athens, School of Medicine, National and Kapodistrian University of Athens, 124 62 Athens, Greece; 3 Microbiology Department, School of Medicine, National and Kapodistrian University of Athens, 115 27 Athens, Greece

**Keywords:** CIED infection, *Coxiella burnetii*, ICD, pacing wire, tricuspid valve, endocarditis

## Abstract

*Coxiella burnetii* is one of the most common causes of blood culture-negative infective endocarditis (IE). However, only a few cases of cardiac implantable electronic devices (CIED) infection have been reported in the literature. Herein, we present a case of CIED-related blood culture-negative infection attributed to *C. burnetii*. A 54-year-old male was admitted to our hospital due to prolonged fatigue, a low-grade fever lasting more than a month, and weight loss. Three years ago, he received an implantable cardiac defibrillator (ICD) as a primary prevention measure against sudden cardiac death. An initial transthoracic and transesophageal echocardiography showed a dilated left ventricle with severely impaired systolic function, while the ventricular pacing wire was inside the right ventricle with a large echogenic mass (2.2 × 2.5 cm) adherent to it. Repeated blood cultures were negative. The patient underwent transvenous lead extraction. A transesophageal echocardiography after the extraction revealed multiple vegetations on the tricuspid valve with moderate to severe valve regurgitation. A surgical replacement of the tricuspid valve was determined after a multidisciplinary heart team approach. Serology tests showed increased IgG antibodies in phase I (1:16,394) and phase II (1:8192), and a definite diagnosis of CIED infection was made based on the serological tests.

## 1. Introduction

*Coxiella burnetii* is one of the most common causes of blood culture-negative infective endocarditis (IE) (second only to prior antibiotic use) in prosthetic and native heart valves [1,2]. However, only a few cases of cardiac implantable electronic device (CIED)-related infections have been reported in the literature [3]. Q fever IE presents a diagnostic challenge in everyday clinical practice and a delayed diagnosis can be fatal if not treated properly. Herein, we present a case of CIED-related blood culture-negative infection attributed to *C. burnetii*.

## 2. Case Report

A 54-year-old male was admitted to our hospital due to prolonged fatigue, a low-grade fever lasting more than a month, and weight loss. He was an active smoker, and his medical history included heart failure with reduced left ventricular ejection fraction (≈30%) due to ischemic heart disease (silent myocardial infarction five years ago and a chronic total occlusion of the left ascending artery), dyslipidemia, and diabetes mellitus type II. He was recently diagnosed (15 days ago) with pulmonary embolism and was under therapeutic-dose anticoagulation therapy with fondaparinux 7.5 mg subcutaneously once daily (OD). Three years ago, he received an implantable cardiac defibrillator (ICD) as a primary prevention measure against sudden cardiac death. He was working as a truck driver and lived in a rural area. No direct animal contact was reported. Before the hospital admission, he had received antibiotic therapy with amoxicillin/clavulanic acid (875 + 125) mg per os twice daily (BID).

**Figure 1:**
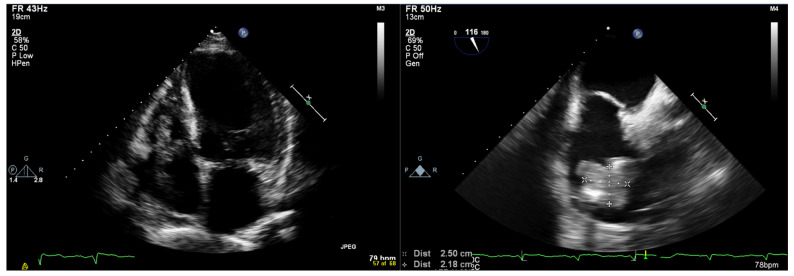
Ventricular pacing wire was inside the right ventricle with a large echogenic mass (2.2 × 2.5 cm) adherent to it. (**Left**) Transthoracic echocardiography study. (**Right**) Transesophageal echocardiography study.

An initial physical examination revealed normal blood pressure (110/60 mm Hg), tachycardia (110 bpm) and a normal oxygen saturation of 96% (FiO_2_ 21%). Heart sounds were normal without murmurs. The laboratory exams showed mild hypochromic microcytic anemia (Hemoglobin = 11 g/dL), mild neutropenia (1.80 × 10⁹/L), thrombocytopenia (37 × 10⁹/L) and an elevated C-reactive protein. A sternal bone marrow aspiration showed supracellular bone marrow and the thrombocytopenia was attributed to an increased peripheral consumption of the platelets due to infection. The electrocardiogram showed sinus rhythm with a QS pattern in leads V1–V3. An initial transthoracic (TTE) and transesophageal echocardiography (TEE) showed a dilated left ventricle with severely impaired systolic function (LVEF of 30%), a normal size and systolic function of the right ventricle, mild aortic, mitral, and tricuspid valve regurgitation, and a small circumferential pericardial effusion. The ventricular pacing wire was inside the right ventricle with a large echogenic mass (2.2 × 2.5 cm) adherent to it (Figure 1, Videos S1 and S2). Our differential diagnosis included vegetation due to CIED-related infection or thrombus. An empiric antibiotic therapy with vancomycin, ceftriaxone and gentamycin was administered to the patient. Repeated blood cultures were negative.

The patient underwent transvenous lead extraction. After the procedure, the patient demonstrated hypoxia and hemodynamic instability. A computed tomography pulmonary angiogram revealed filling defects in the right and left pulmonary arteries (consistent with septic pulmonary embolism), as well as the anterior and inferior vena cava. A transesophageal echocardiography after the extraction revealed multiple vegetations on the tricuspid valve with moderate to severe valve regurgitation. A surgical replacement of the tricuspid valve was determined after a multidisciplinary heart team approach. Serology tests showed increased IgG antibodies in phase I (1:16,394) and phase II (1:8192). According to the recommendations for the diagnosis of CIED infections (the Novel 2019 International CIED Infection Criteria) [4], a definite diagnosis of CIED infection was made. The polymerase chain reaction (PCR) in the formalin-fixed, paraffin-embedded (FFPE) tricuspid valve tissue was negative for *C. burnetti*. A real-time PCR was performed on the DNA extracted from 10 microns thick sections of the fixed and embedded pathology specimen. The sections were deparaffinized and rehydrated using a standard xylene/ethanol/deionized H_2_O protocol. DNA was extracted from the sections using the QIAamp DNA mini kit (Qiagen, Hilden, Germany) and the tissue protocol, according to the manufacturer’s instruction (except for the incubation with proteinase K at 56 °C which was performed overnight). A real-time PCR targeting the *Coxiella burnetii* DNA gyrase subunit A was performed using the *C. burnetii* Genesig real-time PCR kit (Primer Design, Eastleigh, SO53 4DG, UK) on a SteOnePlus real-time PCR instrument (Applied Biosystems, Waltham, MA, USA). PCR products were detected on the FAM channel.

The patient received long-term antibiotic therapy with doxycycline 100 mg twice a day and hydroxychloroquine 600 mg daily. After one month, due to the presence of pancytopenia, the hydroxychloroquine was replaced with levofloxacin 500 mg per os once daily. The patient remained afebrile and had an uneventful recovery with regular follow-up visits for a period of six months.

## 3. Discussion

CIED-related infections have become more common in recent decades [4]. The management of blood culture-negative IE is always challenging, while the detection of an etiological agent is difficult, especially in the case of prior antibiotic use. To our knowledge, although *C. burnetti* is one of the most common causes of IE, there are only three reported cases of CIED-related infection attributed to this bacterium [1,2]. The first two cases were reported by Oteo et al. [2] in 2012. In fact, *C. burnetii* is considered an extremely rare cause of CIED or tricuspid valve endocarditis.

The imaging data should be evaluated by a multidisciplinary team (the Endocarditis Team), as this has been found to have a considerably lower 1-year mortality, from 18.5% to 8.2% [4]. In our patient, vegetation was obvious on the pacing wire from the echocardiography study. However, in cases of high clinical suspicion of CIED-related infection with negative blood cultures, several imaging methods are currently available to evaluate these patients. Although TTE and TEE are the first imaging tools in the assessment of patients with suspected CIED infection, newer imaging methods, such as intracardiac echocardiography (ICE), Fluorine-18 fludeoxyglucose ([18F]FDG) positron emission tomography/computerized tomography (PET/CT) scanning and radiolabeled leucocyte (WBC) scintigraphy, are valuable tools for the diagnosis of CIED-related infection. In particular, if there is a suspicion of CIED-related infection, positive blood cultures, negative TTE and TEE, ICE, [^18^F] FDG PET/CT scanning, radiolabeled WBC scintigraphy or contrast enhanced CT are recommended to establish our diagnosis [4].

Our patient underwent a percutaneous transvenous extraction of the lead. A surgical extraction of the lead was also an option, especially for our patient in whom the vegetation was >20 mm. In particular, percutaneous aspiration of vegetations before and during transvenous lead extraction or, alternatively, surgical extraction may be taken into consideration in patients with systemic infection and lead vegetations of >20 mm [4]. However, this recommendation comes from observational studies, while small case series have reported good short-term outcomes of transvenous lead extraction procedures in patients with large lead vegetation despite a high percentage of pulmonary embolism. Based on the available evidence and the patient’s frailty status, the heart team decided to perform percutaneous transvenous extraction of the lead.

In our case, PCR was performed in FFPE tissue of the tricuspid valve. The PCR results on FFPE tissues could be falsely negative, or *C. burnetii* could be latently present in the tissue and go undetected by PCR [5,6]. In cases of chronic Q fever, the diagnostic value increases significantly when the analysis is performed using fresh specimens [1,7]. The negative result of the PCR in our patient can be attributed to the above reasons. We did not send fresh tricuspid valve tissue for PCR as we already had a definite diagnosis of *C. burnetii* IE according to the current guidelines. Immunofluorescence and fluorescence in situ hybridization have been tested as alternative methods to PCR in the detection of *C. burnetii* [5,6]. However, PCR remains the gold standard of examination for the detection of *C. burnetii* in FFPE or fresh tissues.

Our case report highlights the importance of having a high clinical suspicion for *C. burnetii* in patients with culture-negative CIED-related infections, even in the absence of a positive PCR test. Clinical risk factors, epidemiology, serology and imaging examinations should guide the clinician’s therapeutic decisions.

## Supplementary Materials

The following supporting information can be downloaded at: https://www.mdpi.com/article/10.3390/jcm12082817/s1, Video S1: Transthoracic echocardiography study. Ventricular pacing wire with a large echogenic mass, Video S2: Transesophageal echocardiography study. Ventricular pacing wire with a large echogenic mass.
